# Saturation mutagenesis defines novel mouse models of severe spine deformity

**DOI:** 10.1242/dmm.048901

**Published:** 2021-06-18

**Authors:** Jonathan J. Rios, Kristin Denton, Hao Yu, Kandamurugu Manickam, Shannon Garner, Jamie Russell, Sara Ludwig, Jill A. Rosenfeld, Pengfei Liu, Jake Munch, Daniel J. Sucato, Bruce Beutler, Carol A. Wise

**Affiliations:** 1 Center for Pediatric Bone Biology and Translational Research, Scottish Rite for Children, Dallas, TX 75219, USA; 2 Department of Pediatrics, The University of Texas Southwestern Medical Center, Dallas, TX 75390, USA; 3 McDermott Center for Human Growth and Development, The University of Texas Southwestern Medical Center, Dallas, TX 75390, USA; 4 Department of Orthopaedic Surgery, The University of Texas Southwestern Medical Center, Dallas, TX 75390, USA; 5 Simmons Comprehensive Cancer Center, The University of Texas Southwestern Medical Center, Dallas, TX 75390, USA; 6 Division of Genetic and Genomic Medicine, Nationwide Children's Hospital, Columbus, OH 43205, USA; 7 Center for the Genetics of Host Defense, The University of Texas Southwestern Medical Center, Dallas, TX 75390, USA; 8 Department of Molecular & Human Genetics, Baylor College of Medicine, Houston, TX 77030, USA; 9 Baylor Genetics, Houston, TX 77021, USA; 10 Department of Orthopaedics, Scottish Rite for Children, Dallas, TX 75219, USA

**Keywords:** ENU, N-ethyl-N-nitrosourea, Scoliosis, Kyphosis

## Abstract

Embryonic formation and patterning of the vertebrate spinal column requires coordination of many molecular cues. After birth, the integrity of the spine is impacted by developmental abnormalities of the skeletal, muscular and nervous systems, which may result in deformities, such as kyphosis and scoliosis. We sought to identify novel genetic mouse models of severe spine deformity by implementing *in vivo* skeletal radiography as part of a high-throughput saturation mutagenesis screen. We report selected examples of genetic mouse models following radiographic screening of 54,497 mice from 1275 pedigrees. An estimated 30.44% of autosomal genes harbored predicted damaging alleles examined twice or more in the homozygous state. Of the 1275 pedigrees screened, 7.4% presented with severe spine deformity developing in multiple mice, and of these, meiotic mapping implicated N-ethyl-N-nitrosourea alleles in 21% of pedigrees. Our study provides proof of concept that saturation mutagenesis is capable of discovering novel mouse models of human disease, including conditions with skeletal, neural and neuromuscular pathologies. Furthermore, we report a mouse model of skeletal disease, including severe spine deformity, caused by recessive mutation in *Scube3*. By integrating results with a human clinical exome database, we identified a patient with undiagnosed skeletal disease who harbored recessive mutations in *SCUBE3*, and we demonstrated that disease-associated mutations are associated with reduced transactivation of Smad signaling *in vitro*. All radiographic results and mouse models are made publicly available through the Mutagenetix online database with the goal of advancing understanding of spine development and discovering novel mouse models of human disease.

## Abstract (precis)

**Summary:** We report selected mouse models of spine deformity following mutagenesis across 30% of autosomal genes, results of which are made publicly available to advance understanding of spine development and disease.

## INTRODUCTION

Development of the spinal column is a highly regulated process that begins early in embryogenesis. Postnatal growth and maintenance of a structurally sound spine requires coordinated integration of the vertebrae (skeleton), intervertebral discs, nerves and muscles. Disruption of any of these components may lead to spinal malformations. In humans, scoliosis, defined as a lateral curvature of the spine, is the most common spine deformity and is characterized as idiopathic, congenital, neuromuscular or syndromic (Herring, 2013). Alternatively, kyphosis manifests as an anterior-posterior curvature of the spine, sometimes in combination with scoliosis (so-called kyphoscoliosis). Unlike idiopathic scoliosis, congenital scoliosis can be associated with skeletal malformations or segmentation defects of the vertebrae, and neuromuscular scoliosis is associated with neurological or muscular disease etiologies. Each of these disease types present with different scoliotic deformities and may require different treatment paradigms.

Population-based genome-wide association studies have identified several loci associated with idiopathic scoliosis, and next-generation sequencing studies have elucidated rare mutations underlying congenital scoliosis (Chen et al., 2020; Gao et al., 2007; Khanshour et al., 2018; Kou et al., 2013; Sharma et al., 2011, 2015; Takahashi et al., 2011; Wu et al., 2015). Despite the success of human genetic studies, modeling spine deformities for these loci in mice has proven to be challenging. For example, the correlation of human scoliosis with non-genetic factors, such as the adolescent growth spurt, hormonal influences during adolescence and the distribution of mechanical forces of a bipedal spine, may be difficult to recapitulate in mice or other model systems. Moreover, certain genes implicated in severe forms of scoliosis, such as *TBX6*, may be sub-viable in genetically engineered knockout mouse models (Chapman and Papaioannou, 1998; Wu et al., 2015). Alternative strategies, such as using Cre-inducible conditional knockout mouse models or engineering hypomorphic alleles, have been used successfully to investigate scoliosis-associated genes *in vivo* (Karner et al., 2015; Yang et al., 2019). However, these strategies are limited due to a lack of knowing *a priori* the relevant cell type for conditional models or which alleles may be hypomorphic and allow for viable mouse models of spine deformity in essential genes.

To facilitate discovery of genes required for proper spine development, we performed live-animal radiography to detect severe spine deformity as part of a saturation mutagenesis skeletal screen in mice (Rios et al., 2021; Wang et al., 2015). As proof of concept, we report selected results from 54,497 mice from 1275 pedigrees. As described previously (Wang et al., 2018), estimates of saturation based on the standard of detecting lethal effects in a curated collection of essential genes suggests that 30.44% of all protein-encoding autosomal genes were mutated to a state of detectable hypomorphism and examined in the homozygous state twice or more in this sample of mice. Among the mutations were 5746 putative null alleles in 4320 genes examined twice or more in the homozygous state. Mutations in genes regulating proper skeletal, muscular and nervous system development are all implicated in scoliotic manifestations discovered through our screen. Results from our study may inform human genetic studies, provide novel mouse models of human disease, and advance knowledge of spine development and deformity. As proof of principle, we cross-referenced results from our study with a database of undiagnosed subjects who underwent clinical exome sequencing, and we report a patient with a skeletal syndrome caused by recessive mutations in the *SCUBE3* gene that resulted in reduced heterodimerization and reduced Smad signal transduction. All results and mouse models, including others not reported here, have been made publicly available through the online Mutagenetix database (https://mutagenetix.utsouthwestern.edu).

## RESULTS

### Radiographic screen for spine deformity

**Fig. 1.**
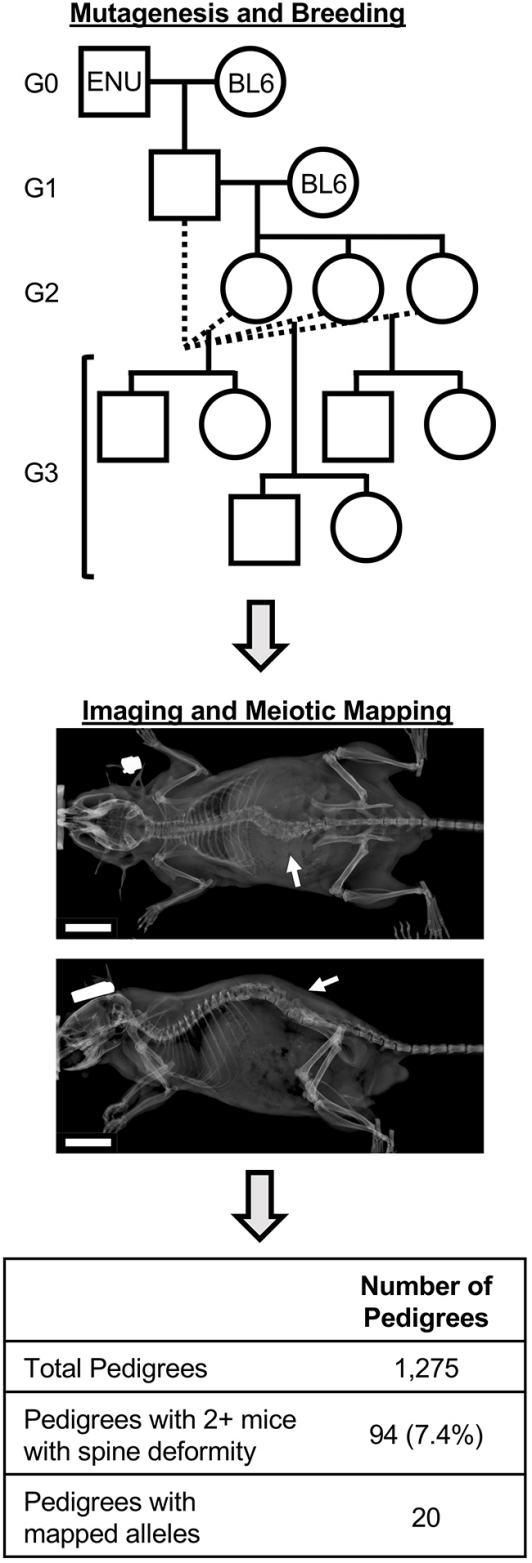
**Schematic of the mutagenesis, breeding and screening strategy.** Mutagenized G0 mice (ENU) are outcrossed to C57BL/6J (BL6) females. G1-generation pups are outcrossed, and subsequent G2-generation mice are backcrossed to their G1 sire to produce pedigrees of G3 mice for screening. Dorsal and lateral radiographs are obtained for all G3 mice, and mice are visually scored as affected or unaffected. Arrows indicate a spine deformity. Automated meiotic mapping tests whether ENU alleles are associated with the spine deformity phenotype after correction for the number of ENU alleles tested in the pedigree. Scale bars: 1 cm.

The breeding scheme for generating and screening mice harboring N-ethyl-N-nitrosourea (ENU)-induced alleles is shown in Fig. 1. C57BL/6J male mice are mutagenized with ENU and outcrossed to non-mutagenized C57BL/6J females, resulting in male pups (termed the G1 generation) heterozygous for germline ENU-induced alleles. G1 male mice are outcrossed to C57BL/6J female mice, and G2 pups are subsequently backcrossed to their G1 sire to produce G3 mice homozygous for the reference allele, heterozygous for the ENU allele, or homozygous for the ENU allele. All G3 mice undergo radiographic screening, including both dorsal and lateral radiography (Fig. 1). Phenotypic scoring is performed from radiographs as either presence or absence of any rigid spine deformity or malformation, such as kyphosis, scoliosis or kinked/curly tail.

ENU-induced alleles are detected by exome sequencing G1 male mice, as described previously (Wang et al., 2015). Nonsynonymous variants identified relative to the C57BL/6J reference genome are genotyped by targeted capture and sequencing of all G2 and G3 mice. Automated meiotic mapping tests the null hypothesis that ENU-induced alleles segregating within the pedigree are not associated with a risk for spine deformity. Significantly associated loci are identified following Bonferroni correction for the number of ENU-induced alleles in the pedigree.

Of the 1275 pedigrees evaluated, 94 (7.3%) pedigrees were identified with at least two mice scored with a spine deformity phenotype. Of these, 20 (20.6%) were mapped to ENU-induced alleles (Fig. 1; Table S1), including 19 recessive and one dominant allele. We sought to evaluate variables limiting detection of significantly associated loci, such as the total number of mice in the pedigree and the total number of affected mice in the pedigree. Pedigrees with mapped alleles had significantly higher numbers of affected mice (Wilcoxon, *P*=1.59e^−5^) compared to unmapped pedigrees (Fig. S1A), and this remained significant among only recessive alleles (Wilcoxon, *P*=4.41e^−5^). Total numbers of mice were unchanged between pedigrees with mapped and unmapped alleles (Fig. S1B). Most (12/19; 63%) pedigrees with mapped recessive alleles had at least 80% penetrance of the spine deformity phenotype among homozygous G3 mice (Table S1).

### Altered skeletal development leading to spine deformity in mice

#### Mouse model and case report of SCUBE3-associated skeletal disease

**Fig. 2.**
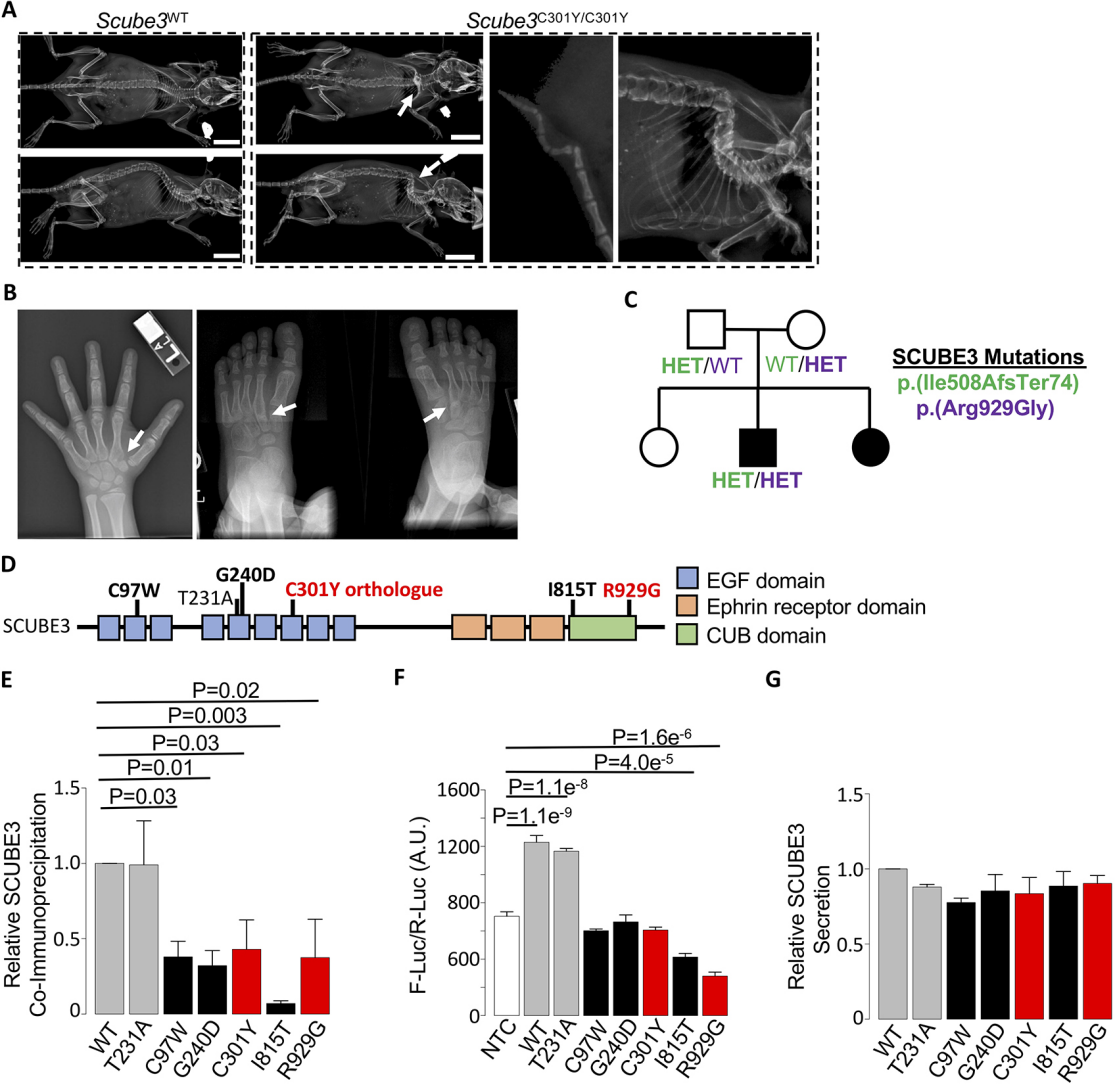
**Mutations in *SCUBE3* are associated with skeletal deformity.** (A) Representative radiographic imaging of spine deformity in 2-month-old female mice homozygous for the *Scube3*^C301Y^ allele compared to a gender-matched littermate homozygous for the reference allele (*Scube3*^WT^). Enlarged radiographs of the kinked tail and spine deformities are shown (right). Arrows indicate location of the deformity. Scale bars: 1 cm. (B) Clinical radiographs of the hand (left) and feet (right) of a male patient with bi-allelic *SCUBE3* mutations. Arrows indicate bone fusions that were evident in both the hands and feet. (C) Pedigree of a family with recessive skeletal disease. Affected individuals are shown with filled symbols. Two mutations were identified by exome sequencing, and genotype results for each mutation are shown for available subjects. Both mutations were confirmed to be compound heterozygous in the affected child by Sanger sequencing (Fig. S2). Circle, female; HET, heterozygous; square, male; WT, no mutation. (D) Schematic of the human SCUBE3 protein, including relative positions of annotated domains. Recently published variants identified in patients with skeletal disease (Lin et al., 2021) are shown in bold black. The human ortholog of the mouse ENU variant and the novel human variant reported here are shown in red. Location of the SCUBE3 p.(Thr231Ala) polymorphism is also shown. (E) Relative normalized quantification of SCUBE3 protein co-immunoprecipitated with SCUBE1. *P*=0.01. (F) The Smad signaling luciferase reporter MDA-scp28 cells were treated with conditioned medium from HEK293T cells expressing wild-type SCUBE3 (WT) and SCUBE3 containing the common polymorphism (T231A) or disease-associated mutations (as indicated in D). An untransfected NTC sample is shown as a negative control. *P*=2.695e^−12^. (G) Relative secretion of Myc-tagged SCUBE3 was evaluated in medium from HEK293 cells transiently expressing wild-type SCUBE3 (WT) and SCUBE3 containing the common polymorphism (T231A) or disease-associated mutations (as indicated in D). *P*=0.32. Data are mean±s.e.m. from three independent experiments. Statistically significant differences were evaluated by ANOVA and, in F, pairwise differences compared to NTC were determined by Dunnett's test (see Materials and Methods). A.U., arbitrary units.

We identified the *Scube3*^C301Y^ allele associated with recessive spine deformity, including severe kyphosis and kinked tail (Fig. 2A). Although the homozygous *Scube3*^C301Y^ allele co-segregated with homozygous *Rhot2*^V604A^ and *Nrde2*^D607E^ alleles, we considered these to be less likely candidates based on the presence of skeletal phenotypes in mice homozygous for a previously reported *Scube3*^N294K^ allele (Fuchs et al., 2016). Additionally, the *Scube3*^C301Y^ allele was predicted to be more damaging by PolyPhen analysis than the *Rhot2*^V604A^ and *Nrde2*^D607E^ alleles.

The human *SCUBE3* gene, encoding the signal peptide, CUB domain, and EGF-like domain containing 3 protein, is highly expressed in osteoblasts (Wu et al., 2004). The SCUBE3 protein was identified as a transforming growth factor-beta (TGF-β) receptor ligand and activator of Smad signaling (Wu et al., 2004, 2011). Proper regulation of the TGF-β signaling cascade is essential for normal skeletal development (Crane and Cao, 2014). Disruption of TGF-β signal transduction leads to defects in endochondral ossification via dysregulation of osteogenesis, which has been studied extensively using germline and conditional mouse models (Wu et al., 2016). Furthermore, disruption of TGF-β signaling affects the crosstalk between bone forming osteoblasts and bone resorbing osteoclasts during bone remodeling (Erlebacher and Derynck, 1996; Filvaroff et al., 1999). Mice homozygous for a *Scube3*^N294K^ allele developed mild phenotypic abnormalities, including shorter hindlimbs, rib defects and auditory abnormalities, and *Scube3*^−/−^ mice were recently shown to develop a postnatal dwarfism that was not apparent during embryonic development (Fuchs et al., 2016; Lin et al., 2021; Xavier et al., 2013).

Although all three Scube genes (*Scube1*-*3*) are implicated in regulating proper skeletal development in mice (Fuchs et al., 2016; Lin et al., 2015; Tu et al., 2008), mutations in any one of three human Scube genes (*SCUBE1-3*) have yet to be associated with human disease. However, during the course of this study, multiple subjects with recessive *SCUBE3* mutations were reported with short stature, oral-facial abnormalities, skeletal abnormalities and, although variable, spine deformity, suggesting a novel *SCUBE3*-associated skeletal disease (Lin et al., 2021). To identify additional patients with skeletal disease potentially attributable to mutations in *SCUBE3*, we queried the Baylor Genetics Laboratory database, which includes nonsynonymous variants identified from clinical exome sequencing and clinician-provided clinical synopses. We sought patients described with skeletal abnormalities, yet remaining without a genetic diagnosis following evaluation by clinical exome sequencing, among whom rare nonsynonymous variants in *SCUBE3* were identified. We identified a now 16-year-old male patient presenting with GI malrotation, hearing loss, short stature, joint hyperextensibility, dilated aortic root with abnormal aortic valve, and oral-facial differences, including cupped ears, high-arched palate and micrognathia (Table 1). Skeletal abnormalities included asymmetric fusion of a pseudoepiphysis of the second metacarpal bilaterally (Fig. 2B). His feet were notable for an unusual fusion of the second metatarsal to the second cuneiform (Fig. 2B). The patient's parents were unaffected; however, an affected sibling was also noted to have similar clinical findings, consistent with a recessive disease presentation in this family (Table 1; Fig. 2C). The male patient previously underwent extensive genetic testing, including microarray analysis, testing for congenital disorders of glycosylation and targeted genetic testing, including *COL1A1*, *COL2A1*, *COL3A1*, *COL11A1*, *COL11A2*, *CDH7*, *TGFBR1*, and *TGFBR2*; however, all testing, including subsequent clinical exome sequencing, was negative. Through the database query, the patient was identified with two novel *SCUBE3* variants, including a predicted loss-of-function frameshift mutation [NM_152753:c.1521_1522insGC, p.(Ile508AlafsTer74)] and a missense mutation [NM_152753:c.2785C>G, p.(Arg929Gly)]. Sanger sequencing performed in the patient and both parents confirmed compound heterozygous inheritance in the affected son; additional testing in the siblings was not available (Fig. 2C; Fig. S2A-C).

**Table 1.**
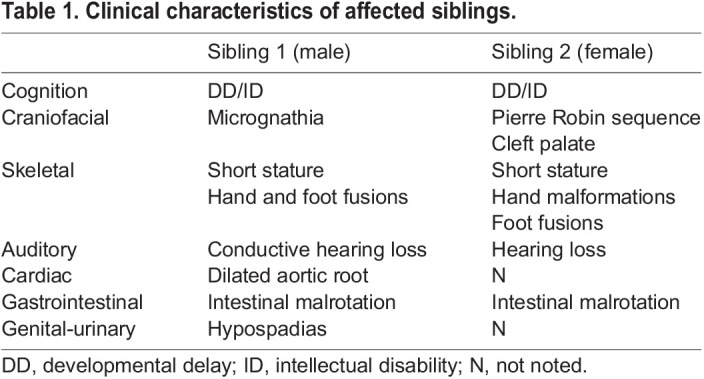
Clinical characteristics of affected siblings.

SCUBE3 was previously shown to heterodimerize with SCUBE1 (Wu et al., 2004), though it remains unclear whether this interaction is required for SCUBE3 function. We performed co-immunoprecipitation experiments to test potential deleterious effects of the novel SCUBE3 p.(Arg929Gly) variant on SCUBE1-SCUBE3 heterodimerization. Results were compared to an allelic series, including to recently reported SCUBE3 variants (Lin et al., 2021), to a common SCUBE3 p.(Thr231Ala) polymorphism (rs79753406) predicted to have normal function, and to the SCUBE3 p.(Cys301Tyr) variant orthologous to our mouse ENU allele associated with severe spine deformity (Fig. 2D). Compared to wild-type and SCUBE3 p.(Thr231Ala) proteins, SCUBE3 containing disease-associated variants showed reduced interaction with SCUBE1 (Fig. 2E; Fig. S2D). These results suggest disease-associated mutations alter the formation of the SCUBE1-SCUBE3 heterodimer.

SCUBE3 has also previously been shown to activate Smad signaling via direct interaction with the TGF-β type II receptor (TGFBR2) (Wu et al., 2011). Consistent with this, clinical features of the index patient reported here resembled Loeys–Dietz syndrome type 2 caused by mutations in *TGFBR2* (Mizuguchi et al., 2004). We tested transactivation of Smad signaling by treating MDA-scp28 cells with conditioned medium from cells expressing recombinant SCUBE3 protein. Conditioned medium from cells expressing the wild-type or SCUBE3 p.(Thr231Ala) protein readily activated Smad signaling, whereas conditioned medium from cells expressing disease-associated SCUBE3 variants failed to activate Smad signaling (Fig. 2F). All samples showed similar levels of SCUBE3 secretion into the conditioned media (ANOVA, *P*=0.32), suggesting differences in transactivation were not due to differences in SCUBE3 secretion (Fig. 2G; Fig. S2D). These results demonstrate disease-associated *SCUBE3* missense mutations are loss-of-function alleles causing *SCUBE3*-associated skeletal disease.

#### Mouse model of Hes7-associated spine deformity

**Fig. 3.**
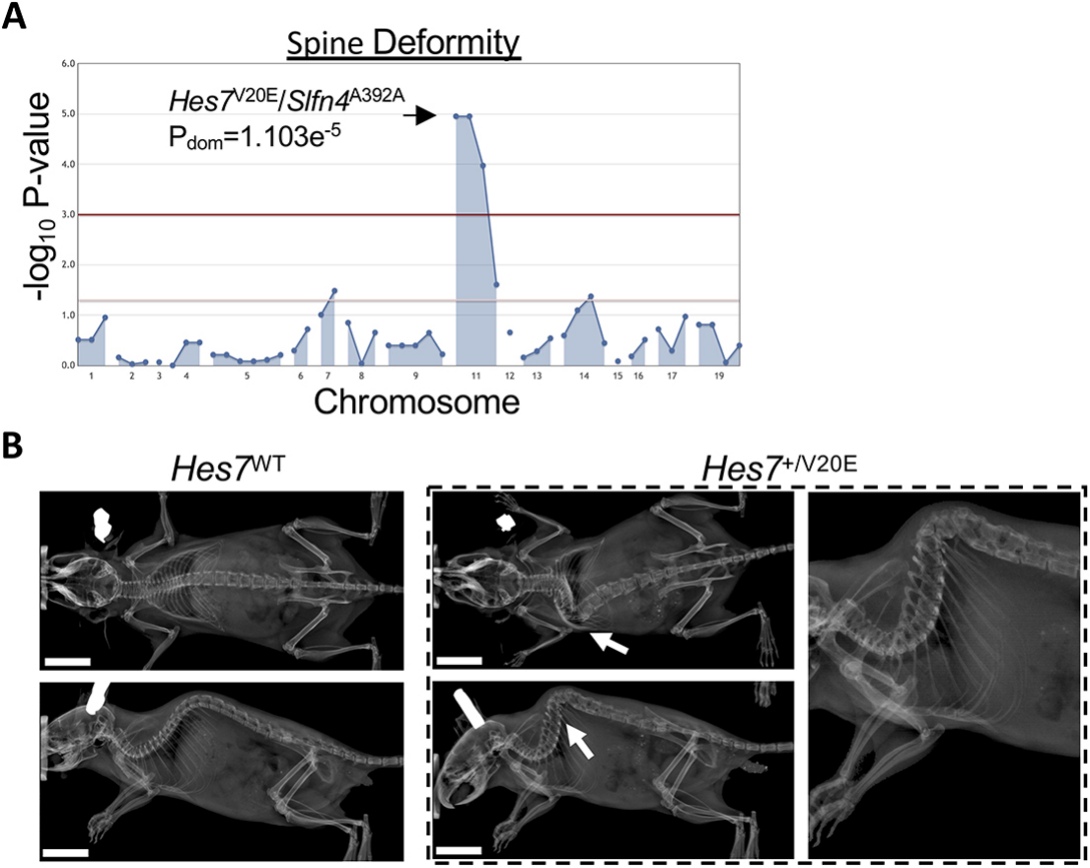
**Mouse model of a dominant *Hes7*-associated spine deformity.** (A) Manhattan plot showing statistical significance following automated meiotic mapping of ENU-induced alleles with a dominant spine deformity phenotype. The *Hes7*^V20E^ allele was significantly associated with spine deformity in heterozygous mice. No homozygous mice were detected. (B) Representative radiographic images of the severe spine deformity in 3-month-old female mice heterozygous for the ENU allele (*Hes7^+/^*^V20E^) compared to a gender-matched littermate homozygous for the reference allele (*Hes7*^WT^). Arrows indicate the location of the deformity. Scale bars: 1 cm.

During early embryonic development, the paraxial mesoderm gives rise to segmented bodies within the pre-somitic mesoderm and, ultimately, to the components of the vertebrate spinal column. The segmented bodies are formed through an intricate cyclical wave of gene regulation (i.e. segmentation clock), including the hairy and enhancer of split (HES)/HES-related (HER) family of transcription factors (Hubaud and Pourquie, 2014). Proper timing of the segmentation clock proceeds through feedback inhibition that controls Notch signaling periodicity. Disruption of the segmentation clock during early development causes various recognizable phenotypes, such as the spondylocostal dysostoses in humans. Spondylocostal dysostosis type 4, in which patients present with a variety of skeletal abnormalities, including spine and vertebral malformations, is caused by recessive loss-of-function mutations in the *HES7* gene (Sparrow et al., 2008, 2010). *Hes7* knockout mice died shortly after birth, whereas some heterozygous *Hes7*^+/−^ mice displayed kinked tails but no other significant skeletal abnormalities (Bessho et al., 2001). We detected significant association of severe spine deformities with the heterozygous *Hes7*^V20E^ allele, which co-segregated with a synonymous mutation in *Slfn4* (Fig. 3A,B). A total of 15/21 (71%) heterozygous mice developed severe kyphoscoliosis with a sharp vertebral angulation resembling human patients with spine deformity associated with segmentation defects, and, of these, six also developed kinked tails. The *Hes7*^V20E^ variant is located at the beginning of the basic-helix-loop-helix domain of the protein and may disrupt DNA binding, which is necessary for transcriptional feedback repression as part of the segmentation clock during mouse somitogenesis (Bessho et al., 2001). Interestingly, no additional skeletal abnormalities were observed in *Hes7*^+/V20E^ mice. As the mice described here develop a more severe spine deformity compared to *Hes7*^+/−^ mice (Bessho et al., 2001), it remains to be determined whether the *Hes7*^V20E^ allele is loss of function or whether it has a dominant-negative effect on Hes7 transcription factor feedback signaling *in vivo*.

### Neurogenic spine deformities in mice

#### Disruption of peripheral nervous system development

**Fig. 4.**
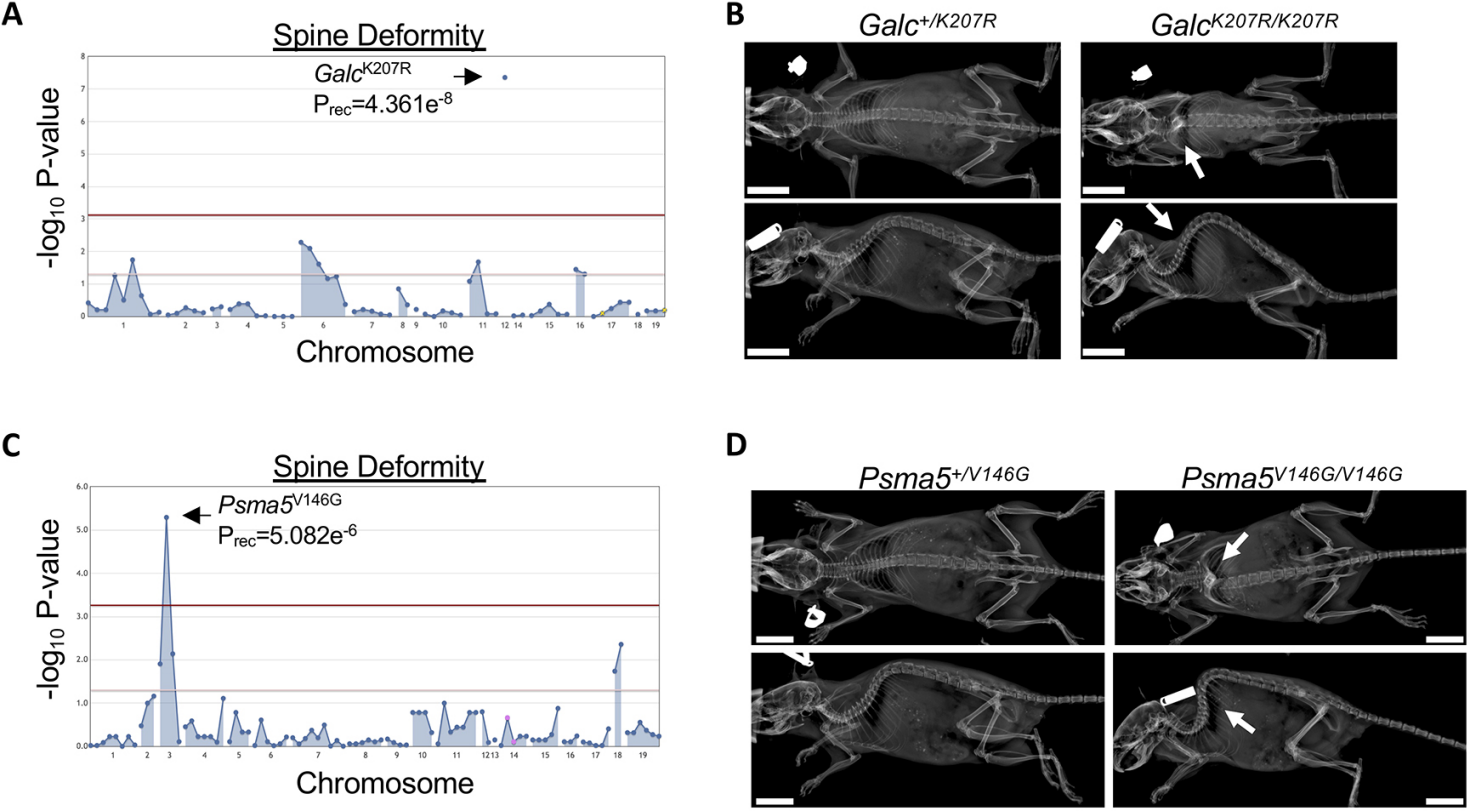
**Neurogenic spine deformity.** (A) Manhattan plot showing statistical significance of the *Galc*^K207R^ allele following automated meiotic mapping of ENU-induced alleles with a recessive spine deformity phenotype. (B) Representative radiographic images of 2-month-old female mice heterozygous for the ENU allele (*Galc*^+/K207R^) or homozygous for the ENU allele (*Galc*^K207R/K207R^). Homozygous mice were smaller and developed a rigid thoracic kyphosis phenotype. Arrows indicate the location of the deformity. (C) Manhattan plot showing statistical significance of the *Psma5*^V146G^ allele following automated meiotic mapping of ENU-induced alleles with a recessive spine deformity phenotype. (D) Representative radiographs of 5-month-old female mice heterozygous for the ENU allele (*Psma5^+/^*^V146G^) or homozygous for the ENU allele (*Psma5*^V146G/V146G^). Arrows indicate the location of the deformity. Scale bars: 1 cm.

Some inborn errors of metabolism, such as lysosomal storage diseases, may manifest as neurodegenerative disease (Onyenwoke and Brenman, 2015). Krabbe disease is caused by recessive mutations in the *GALC* gene, which encodes galactosylceramidase, a lysosomal enzyme that hydrolyzes galactolipids prevalent in myelin of the central and peripheral nervous systems (Sakai et al., 1994; Wenger et al., 2000). Though the age of onset may vary, the majority of patients with Krabbe disease present during infancy with spasticity and developmental delay, leading to death by 2 years of age (Tappino et al., 2010). Among patients with infantile-onset Krabbe disease, most develop scoliosis by 2 years of age (Beltran-Quintero et al., 2019). Loss of Galc activity has been described in the ‘twitcher’ mouse line, which develops early-onset tremor, weakness, late-onset kyphosis and wasting with death by 3 months of age (Duchen et al., 1980; Kobayashi et al., 1980). We identified significant association between the *Galc*^K207R^ allele and recessive spine deformity (Fig. 4A). All six homozygous mice displayed rigid cervicothoracic kyphosis (Fig. 4B) and wasting. The *Galc*^K207R^ variant is located within an alpha-helix of the central triosephosphate isomerase (TIM) barrel domain located at the inner core of the Galc protein, near the Galc^E198^ catalytic residue involved in binding galactose (Deane et al., 2011). These results suggest that the Galc^K207R^ variant results in loss of Galc protein function, possibly through altering substrate binding.

#### Proteasome dysfunction

The 20S proteasome is a multimeric complex composed of seven alpha (PSMA1-7) and seven beta (PSMB1-7) subunits (Demartino and Gillette, 2007). Together with the PA700 regulatory component, the 20S protease degrades polyubiquitinated proteins. The genes encoding alpha and beta subunits of the 20S proteasome are highly conserved in humans, with high intolerance to loss-of-function mutations (Karczewski et al., 2020). Recently, a homozygous missense mutation in *PSMB1* (*PSMB1*^Y103H^) was identified in a consanguineous family presenting with short stature, microcephaly, neurocognitive delay and other manifestations (Ansar et al., 2020). The PSMB1^Y103^ residue was predicted to interact with the PSMA5 subunit, and the PSMB1^Y103H^ variant disrupted the formation and activity of the 20S proteasome in transient transfection assays. In mice, 20S proteasome subunits are largely unstudied; however, we detected significant association with spine deformity in mice homozygous for the *Psma5*^V146G^ allele (Fig. 4C,D). These mice also variably developed ataxia and a wobbling gait (P_REC_=1.15e^−6^; Movie 1), suggestive of a neurologic deficit, and were significantly smaller (Fig. S3). It remains to be determined whether the *Psma5*^V146G^ allele reported here is a complete loss of function or hypomorphic, as *Psma5* is likely an essential gene, and knockout mice have not been characterized.

### Mouse models of neuromuscular disease

#### Myotonic dystrophy

**Fig. 5.**
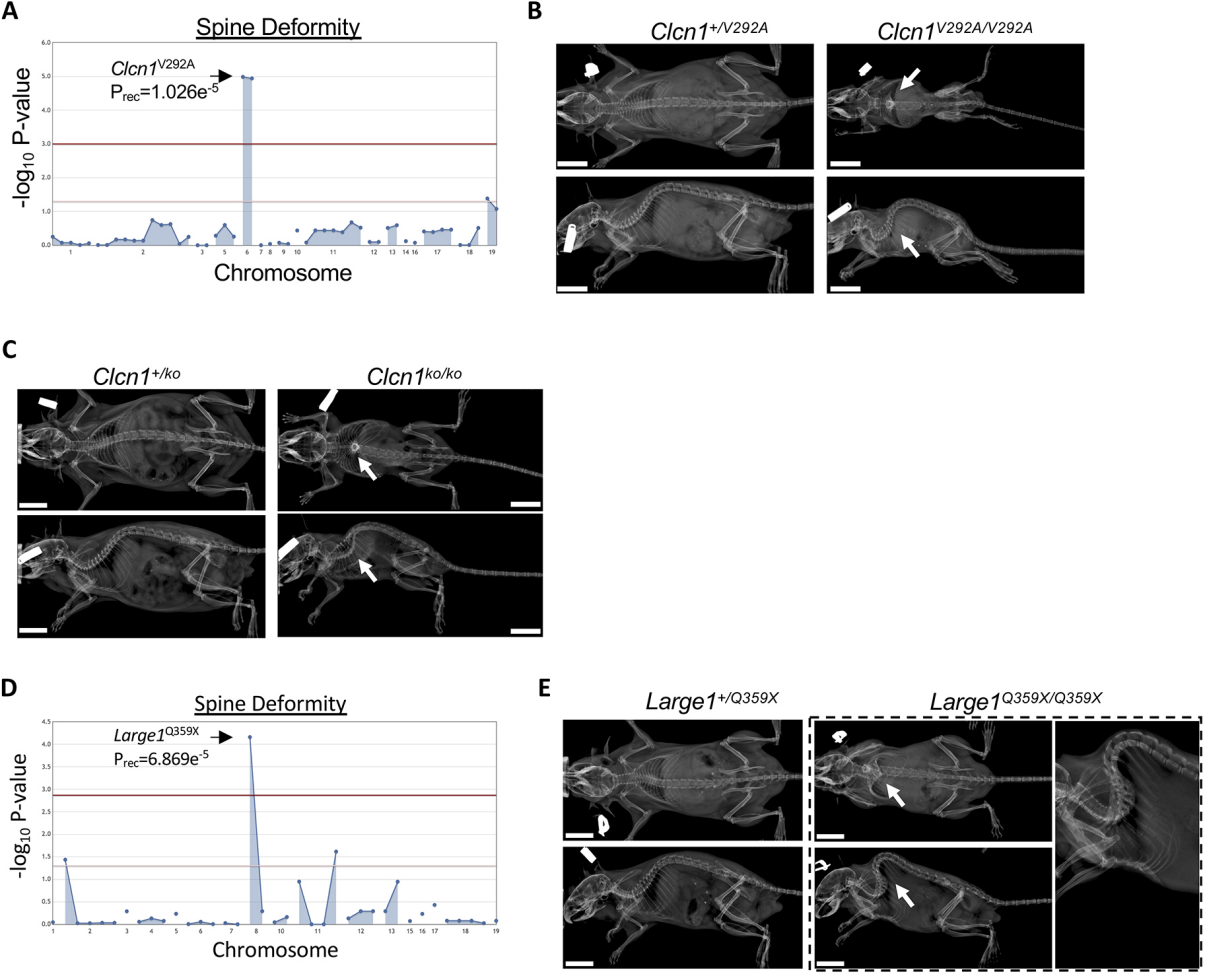
**Neuromuscular spine deformity.** (A) Manhattan plot showing statistical significance of the *Clcn1*^V292A^ allele following automated meiotic mapping of ENU-induced alleles with a recessive spine deformity phenotype. (B) Representative radiographs of 2-month-old male mice heterozygous for the ENU allele (*Clcn1^+/^*^V292A^) or homozygous for the ENU allele (*Clcn1*^V292A/V292A^). Arrows indicate the location of the deformity. (C) Representative radiographs of 5-month-old male CRISPR-engineered mice heterozygous for the p.(Phe337GlyfsTer4) mutation (*Clcn1^+/ko^)* or homozygous for the mutation (*Clcn1*^ko/ko^). Arrows indicate the location of the deformity. (D) Manhattan plot showing statistical significance of the *Large1*^Q359X^ allele following automated meiotic mapping of ENU-induced alleles with a recessive spine deformity phenotype. (E) Representative radiographs of 4-month-old male mice heterozygous for the ENU allele (*Large1^+/^*^Q359X^) or homozygous for the allele (*Large1*^Q359X/Q359X^). Arrows indicate the location of the deformity. Scale bars: 1 cm.

Neuromuscular conditions, such as muscular or myotonic dystrophies, present an increased risk for spine deformities. For example, Becker-type myotonic dystrophy is a skeletal muscle disorder caused by recessive mutations in the *CLCN1* gene, which encodes the chloride voltage-gated channel 1 protein (Lorenz et al., 1994). In contrast, dominant-negative mutations in the same gene cause Thomsen-type myotonic dystrophy, though the recessive Becker type is more common and more severe. Both diagnoses present with a general inability to relax skeletal muscles throughout the body, and some patients with *CLCN1* deficiency have been noted to develop spine deformity (Skalova et al., 2013). The CLCN1 homodimer functions as a chloride ion channel chiefly responsible for opposing excitatory signals in resting skeletal muscle. In mice, a spontaneous *Clcn1* mutant line (*Clcn1*^adr^) was described with myotonia, muscle weakness, reduced growth and brittle bones (Watkins and Watts, 1984). We identified a significant association of the homozgygous *Clcn1*^V292A^ allele with spine deformity (Fig. 5A,B). The *Clcn1*^V292A^ allele co-segregated with a missense allele in the olfactory receptor 13 gene (*Olfr13*^Y59H^); however, the phenotypic resemblance of these mice with the *Clcn1*^adr^ line and the lack of any published characterization of this olfactory receptor led us to implicate the *Clcn1*^V292A^ allele in these mice. Similar to *Clcn1*^adr^ mice, homozygous *Clcn1*^V292A^ mice were noticeably smaller. It is notable that a human mutation (*CLCN1*^E291K^) in the residue adjacent to the orthologous human *CLCN1*^V292^ position was reported in three unrelated families with recessive myotonic dystrophy (Meyer-Kleine et al., 1995). To validate this mouse phenotype, we engineered *Clcn1* knockout mice using CRISPR/Cas9. Knockout mice homozygous for a predicted frameshift mutation, p.(Phe337GlyfsTer4), developed significant spine deformity and reduced growth (Fig. 5C; Table S1), suggesting the *Clcn1*^V292A^ allele is loss of function.

#### Muscular dystrophy

Dystroglycanopathies represent a subset of congenital muscular dystrophies, including Walker–Warburg syndrome and muscle-eye-brain disease. Both are among the most severe of the dystroglycanopathies and are caused by recessive mutations in *LARGE1*, encoding the LARGE xylosyl- and glucuronyltransferase 1 protein (Clement et al., 2008; van Reeuwijk et al., 2007). LARGE1 functions to glycosylate α-dystroglycan, an integral component of the skeletal muscle extracellular matrix (Inamori et al., 2012). α-dystroglycan binds to β-dystroglycan, a myofiber membrane protein that itself binds intracellular dystrophin (Martin, 2003). Disruption of different components of this complex results in several muscular dystrophies. Mice lacking *Large1*, as in the spontaneous *myd* mouse line, show reduced survival, reduced growth and kyphoscoliosis (Grewal et al., 2001; Lane et al., 1976). We identified a significant association of the *Large1*^Q359X^ nonsense allele with recessive spine deformity (Fig. 5D,E). Interestingly, the *Large1*^Q359X^ allele was not significantly associated with postnatal lethality. The variant is located in the middle of the first glycosyltransferase domain and is predicted to result in complete loss of function.

## DISCUSSION

Discovering the genetic causes of human spine deformities has been elusive until recently, due, in part, to technological advances, such as genome-wide association studies and next-generation sequencing (Grauers et al., 2016; Wise et al., 2020). Understanding the genetic and mechanistic basis of human spinal disorders may be accelerated by forward genetic screens in model systems. To identify novel genetic mouse models of spine deformity, we performed a live-animal radiography screen in 54,497 mice collectively harboring predicted damaging or loss-of-function ENU-induced mutations in at least two homozygous mice across 30.44% of autosomal genes. ENU mutagenesis in mice provides unique advantages. First, the saturation mutagenesis approach employed here enables high-throughput generation and testing for associated phenovariance with predicted deleterious and loss-of-function alleles throughout the mouse genome (Wang et al., 2018; Wang et al., 2015). Second, ENU mutagenesis introduces single-base variants typically resulting in missense alterations that may discover hypomorphic effects (Arnold et al., 2012). Third, though less frequently, introduction of missense alleles may disrupt specific protein functions that result in detectable phenotypes not evident in knockout mouse models. Our results demonstrate the feasibility of detecting severe spine deformities by radiographic imaging within a high-throughput saturation mutagenesis screen in mice. Although our approach enables detection of spine deformities that may be missed with observation alone (i.e. kinked or curly tails), the relatively low percentage of pedigrees presenting with spine deformity suggest that challenges remain. For example, limitations from subjective radiographic evaluations may necessitate larger pedigrees to confidently map disease-causing ENU alleles or sexually dimorphic alleles. A recent saturation mutagenesis screen in zebrafish reported similarly low occurrence of pedigrees with spine deformity and with mapped alleles (Gray et al., 2020), suggesting such challenges may not be unique to mouse or other quadruped species. Although selected examples are presented here, results from the entire skeletal screen are deposited in the public online Mutagenetix database, which complements ongoing efforts of the IMPC (mousephenotype.org) to advance discovery of genes required for proper spine development in mice and, potentially, novel mouse models of human disease.

By integrating results with a clinical exome database of undiagnosed patients, we report a patient with bi-allelic *SCUBE3* mutations presenting with skeletal abnormalities. The implication of *SCUBE3* as a skeletal disease-causing gene was supported by our identification of mice homozygous for the *Scube3*^C301Y^ allele developing severe skeletal defects. In the course of this study, multiple patients were reported with recessive *SCUBE3* mutations presenting with skeletal and non-skeletal manifestations similar to the index patient reported here (Lin et al., 2021). Using two independent experimental assays, we show that missense variants identified in our mouse model and the undiagnosed patient reported here, as well as other missense variants recently reported (Lin et al., 2021), abrogated the interaction between SCUBE3 and SCUBE1 and reduced transactivation of Smad signaling. These results demonstrate that the characterization of novel mouse models may inform as-yet undiscovered rare human genetic diagnoses when integrated with patient-based exome sequencing studies.

Results from our study also suggest novel mouse models of human Mendelian skeletal diseases may be readily identified from the Mutagenetix database. As proof of concept, we present novel mouse models for multiple known human diseases representing different developmental etiologies, including segmentation defects (*Hes7*), leukodystrophy (*Galc*) and neuromuscular disease (*Clcn1* and *Large1*). Though selected examples of severe spine deformity are presented here, the Mutagenetix database facilitates discovery of, and public access to, ENU-induced alleles and CRISPR-generated mouse lines associated with a spectrum of severe and mild (i.e. kinked tail) spine deformity phenotypes.

Other mutagenesis efforts have been undertaken to identify alleles associated with spine deformity in non-rodent model systems, such as zebrafish (Gray et al., 2020; Henke et al., 2017). Such large-scale mutagenesis efforts can rapidly resolve causal alleles with the integration of next-generation sequencing (Andrews et al., 2012; Wang et al., 2015). Furthermore, *in vivo* gene editing using CRISPR/Cas9 to simultaneously engineer knockout and allele-specific knock-in models provides for rapid confirmation of suspected disease-causing alleles. By integrating with ongoing large-scale human genome sequencing efforts, *in vivo* saturation mutagenesis screens have the potential to advance discovery of novel rare and ultra-rare human disease-causing genes.

## MATERIALS AND METHODS

### Skeletal mouse screen

The mutagenesis and breeding strategy have been described previously (Wang et al., 2018, 2015). Briefly, male mice (designated G0 generation) were mutagenized with ENU and outcrossed to C57BL/6J female mice. Transmitting ENU-induced alleles were detected in the resulting G1 male mice by exome sequencing. G1 male mice were outcrossed to C57BL/6J females, resulting in G2 mice heterozygous for ENU-induced alleles. Female G2 mice were backcrossed to their G1 sire, and all G2 and resulting G3 mice were genotyped by high-throughput targeted capture and next-generation sequencing.

All G3 mice were screened for spine deformity by live-animal radiography. Mice were anesthetized using inhaled isoflurane and imaged using a Faxitron UltraFocusDXA instrument. Spine deformities were scored as a binary variable by visualizing dorsal and lateral X-ray images. Following phenotype analysis, automated meiotic mapping identified ENU-induced alleles associated with the spine deformity phenotype using dominant and recessive inheritance models (Wang et al., 2015). All procedures were approved by the University of Texas Southwestern Medical Center Institutional Animal Care and Use Committee.

### Automated meiotic mapping of ENU alleles

ENU alleles were genotyped in G2 and G3 mice using a targeted capture and massively parallel sequencing approach. Genotypes of G2 and G3 mice were integrated with skeletal phenotypes, and the Linkage Analyzer tool tested the null hypothesis that each ENU allele within a pedigree has no association with the spine deformity phenotype using dominant and recessive models, as described previously (Wang et al., 2015). Statistical significance was assessed using logistic regression and *P*-values are reported using Wald tests. Results were visualized with Manhattan plots, and significantly associated alleles were those exceeding Bonferroni correction for the number of ENU alleles tested in the pedigree.

### Generation of Clcn1 knockout mice

*Clcn1* mutant mice were engineered using CRISPR/Cas9 as described previously (Ran et al., 2013). Female C57BL/6J mice were superovulated by injection of 6.5 U pregnant mare serum gonadotropin (Millipore), followed by injection of 6.5 U human chorionic gonadotropin (Sigma-Aldrich) 48 h later. The superovulated mice were subsequently mated overnight with C57BL/6J male mice. The following day, fertilized eggs were collected from the oviducts and *in vitro*-transcribed Cas9 mRNA (50 ng/μl) and *Clcn1* small base-pairing guide RNA (50 ng/μl; 5′-GTTCAGAACGAATTTCCGAA-3′) were injected into the cytoplasm or pronucleus of the embryos. The injected embryos were cultured in M16 medium (Sigma-Aldrich) at 37°C in 5% CO_2_. For the production of mutant mice, two-cell-stage embryos were transferred into the ampulla of the oviduct (10-20 embryos per oviduct) of pseudopregnant Hsd:ICR (CD-1) female mice (Harlan Laboratories).

Founders were genotyped by Sanger sequencing. Mice carrying a predicted loss-of-function allele, p.(Phe337GlyfsTer4), were mated to C57BL/6J mice. Subsequent pups harboring heterozygous loss-of-function alleles were intercrossed, and resulting pups underwent skeletal screening. All mice were genotyped by Sanger sequencing by amplifying genomic DNA using the following primers: F, 5′-GTCTTGGTGGAGGTGGTATGAAA-3′, and R, 5′-ATGACTTGGCGATGCAGATAAAC-3′. PCR amplicons were sequenced using the following primers: F, 5′-AAATAGGGGTGATGGGAAGG-3′, and R, 5′-CGAAAACAGCTCCCAAGAAC-3′ (Fig. S4).

### Exome sequencing and analysis

Clinical exome sequencing was performed as described previously (Yang et al., 2014). Briefly, sequencing libraries were generated following capture using the VCRome version 2.1 capture system. Paired-end sequencing was performed using the Illumina HiSeq system with 100 base pair reads, resulting in mean >100× coverage of targeted bases. Sequence reads were analyzed using software designed and implemented in the clinical laboratory. Variants in *SCUBE3* were confirmed by Sanger sequencing (Fig. S2, Table S2). As per Institutional guidelines, informed consent was not obtained nor required for the case report included here.

### Co-immunoprecipitation

The *SCUBE1* coding sequence (NM_173050.3) was cloned into pcDNA3.1 introducing an N-terminal hemagglutinin (HA) tag, and the *SCUBE3* coding sequence (NM_152753.2) was cloned into pcDNA3.1 introducing a C-terminal MYC tag. Site-directed mutagenesis (Takara, Mountain View, CA, USA) was used to introduce the following point mutations: SCUBE3 p.(Thr231Ala); SCUBE3 p.(Cys97Trp); SCUBE3 p.(Gly240Asp); SCUBE3 p.(Cys301Tyr); SCUBE3 p.(Ile815Thr); and SCUBE3 p.(Arg929Gly). HA-tagged *SCUBE1* was co-transfected with MYC-tagged *SCUBE3* using FuGene HD (Promega, Madison, WI, USA) into HEK-293 T cells cultured in Dulbecco's modified Eagle medium (DMEM) with 10% fetal bovine serum (FBS) and antibiotic. After 48 h, cells were incubated with RIPA buffer (Santa Cruz Biotechnology, Dallas, TX, USA), scraped from the well, incubated on ice and then pelleted at 12,000 ***g***. Cell lysate (10 µl) was used for western blotting (input).

Pure Proteome Protein A/G Mix Magnetic Beads (Sigma-Aldrich, St. Louis, MO, USA) were centrifuged at 1000 ***g*** for 30 s at 4°C, then incubated with 900 µl cell lysate and 10 µg HA-antibody (Sigma-Aldrich, St. Louis, MO, USA) at 4°C overnight with mixing. After incubation, the beads were pelleted by centrifugation at 1000 ***g*** for 30 s at 4°C. The pellet was washed three times with RIPA buffer. After, the pellet was resuspended in 20 µl Electrophoresis Sample Buffer (Santa Cruz Biotechnology, Dallas, TX, USA). The beads were boiled for 5 min, centrifuged at 1000 ***g*** for 30 s, and used for western blot analysis. Primary antibodies used for western blotting were anti-HA (1:1000; polyclonal, Sigma-Aldrich, St. Louis, MO, USA, H6908) and anti-Myc (1:1000; clone 9E10; Sigma-Aldrich, OP10). Secondary antibodies were IRDye goat anti-mouse IgG (1:5000; Li-Cor, 926-68070) and goat anti-rabbit IgG (1:5000; Li-Cor, 926-32211).

Western blots from three replicate experiments were quantified using ImageJ. For each experiment, the amount of SCUBE3 and SCUBE1 protein used for immunoprecipitation (by volume) was calculated based on the input sample, and the proportion of protein recovered following immunoprecipitation was calculated. The proportion of SCUBE3 protein recovered after immunoprecipitation was normalized to the proportion of SCUBE1 protein recovered after immunoprecipitation. These values were evaluated for statistically significant differences using ANOVA, and, to account for inter-experiment variation, the experimental replicate was included as a covariate. ANOVA *P*-values for each SCUBE3 variant are shown. For plotting, each variant SCUBE3 was then normalized to the SCUBE3^WT^ control from the same experiment, and the mean and s.e.m. across the three replicate experiments were plotted.

### Smad activation assays

The coding sequence of human *SCUBE3* (NM_152753.2) was cloned into pcDNA3.1 introducing an N-terminal hemagglutinin (HA) tag. Site-directed mutagenesis was used to introduce the following point mutations: SCUBE3 p.(Thr231Ala); SCUBE3 p.(Cys97Trp); SCUBE3 p.(Gly240Asp); SCUBE3 p.(Cys301Tyr); SCUBE3 p.(Ile815Thr); and SCUBE3 p.(Arg929Gly). Each clone was transfected into HEK-293T cells, and 48 h later, conditioned medium was harvested and concentrated. The *Smad* reporter cell line MDA-scp28 was treated with conditioned medium to quantify transactivation of Smad signaling (Korpal et al., 2009). Briefly, 50,000 MDA-scp28 cells/well were plated in a 96-multiwell plate in high glucose DMEM supplemented with 10% FBS and 100 U/ml penicillin/streptomycin. Cells were not tested for contamination. MDA-scp28 cells were serum-starved for 24 h then treated with conditioned medium for 20 h. Luciferase activity was detected using a Dual Luciferase kit (Promega, Madison, WI, USA, E1910), following the manufacturer's instructions. Firefly luciferase activity was normalized by the *Renilla* luciferase activity (ratio F-Luc/R-Luc). Statistically significant differences were determined by one-way ANOVA and pairwise differences compared to no template control (NTC) were determined by Dunnett's test.

For testing SCUBE3 secretion, 5 ml of medium was concentrated to 200 µl by centrifugation in Amicon Ultra-4 filters (Sigma-Aldrich), then treated with an Aurum serum protein mini kit (Bio-Rad). For each sample, 60 µl was evaluated by western blotting using an anti-Myc antibody (1:1000; clone 9E10; Sigma-Aldrich, OP10). Western blots from three replicate experiments were quantified using ImageJ. These values were evaluated for statistically significant differences using ANOVA, and, to account for inter-experiment variation, the experimental replicate was included as a covariate. For plotting, each clone was normalized to the SCUBE3^WT^ control from the same experiment, and the mean and s.e.m. across the three replicate experiments were plotted.

## Supplementary Material

Supplementary information

## References

[DMM048901C1] Andrews, T. D., Whittle, B., Field, M. A., Balakishnan, B., Zhang, Y., Shao, Y., Cho, V., Kirk, M., Singh, M., Xia, Y. et al. (2012). Massively parallel sequencing of the mouse exome to accurately identify rare, induced mutations: an immediate source for thousands of new mouse models. *Open Biol.* 2, 120061. 10.1098/rsob.120061 22724066 PMC3376740

[DMM048901C2] Ansar, M., Ebstein, F., Ozkoc, H., Paracha, S. A., Iwaszkiewicz, J., Gesemann, M., Zoete, V., Ranza, E., Santoni, F. A., Sarwar, M. T. et al. (2020). Biallelic variants in PSMB1 encoding the proteasome subunit beta6 cause impairment of proteasome function, microcephaly, intellectual disability, developmental delay and short stature. *Hum. Mol. Genet.* 29, 1132-1143. 10.1093/hmg/ddaa032 32129449

[DMM048901C3] Arnold, C. N., Barnes, M. J., Berger, M., Blasius, A. L., Brandl, K., Croker, B., Crozat, K., Du, X., Eidenschenk, C., Georgel, P. et al. (2012). ENU-induced phenovariance in mice: inferences from 587 mutations. *BMC Res. Notes* 5, 577. 10.1186/1756-0500-5-577 23095377 PMC3532239

[DMM048901C4] Beltran-Quintero, M. L., Bascou, N. A., Poe, M. D., Wenger, D. A., Saavedra-Matiz, C. A., Nichols, M. J. and Escolar, M. L. (2019). Early progression of Krabbe disease in patients with symptom onset between 0 and 5 months. *Orphanet J. Rare Dis.* 14, 46. 10.1186/s13023-019-1018-4 30777126 PMC6378723

[DMM048901C5] Bessho, Y., Sakata, R., Komatsu, S., Shiota, K., Yamada, S. and Kageyama, R. (2001). Dynamic expression and essential functions of Hes7 in somite segmentation. *Genes Dev.* 15, 2642-2647. 10.1101/gad.930601 11641270 PMC312810

[DMM048901C7] Chapman, D. L. and Papaioannou, V. E. (1998). Three neural tubes in mouse embryos with mutations in the T-box gene Tbx6. *Nature* 391, 695-697. 10.1038/35624 9490412

[DMM048901C8] Chen, W., Lin, J., Wang, L., Li, X., Zhao, S., Liu, J., Akdemir, Z. C., Zhao, Y., Du, R., Ye, Y. et al. (2020). TBX6 missense variants expand the mutational spectrum in a non-Mendelian inheritance disease. *Hum. Mutat.* 41, 182-195. 10.1002/humu.23907 31471994 PMC7061259

[DMM048901C9] Clement, E., Mercuri, E., Godfrey, C., Smith, J., Robb, S., Kinali, M., Straub, V., Bushby, K., Manzur, A., Talim, B. et al. (2008). Brain involvement in muscular dystrophies with defective dystroglycan glycosylation. *Ann. Neurol.* 64, 573-582. 10.1002/ana.21482 19067344

[DMM048901C10] Crane, J. L. and Cao, X. (2014). Bone marrow mesenchymal stem cells and TGF-beta signaling in bone remodeling. *J. Clin. Invest.* 124, 466-472. 10.1172/JCI70050 24487640 PMC3904610

[DMM048901C11] Deane, J. E., Graham, S. C., Kim, N. N., Stein, P. E., McNair, R., Cachon-Gonzalez, M. B., Cox, T. M. and Read, R. J. (2011). Insights into Krabbe disease from structures of galactocerebrosidase. *Proc. Natl. Acad. Sci. USA* 108, 15169-15173. 10.1073/pnas.1105639108 21876145 PMC3174575

[DMM048901C12] Demartino, G. N. and Gillette, T. G. (2007). Proteasomes: machines for all reasons. *Cell* 129, 659-662. 10.1016/j.cell.2007.05.007 17512401

[DMM048901C13] Duchen, L. W., Eicher, E. M., Jacobs, J. M., Scaravilli, F. and Teixeira, F. (1980). Hereditary leucodystrophy in the mouse: the new mutant twitcher. *Brain* 103, 695-710. 10.1093/brain/103.3.695 7417782

[DMM048901C14] Erlebacher, A. and Derynck, R. (1996). Increased expression of TGF-beta 2 in osteoblasts results in an osteoporosis-like phenotype. *J. Cell Biol.* 132, 195-210. 10.1083/jcb.132.1.195 8567723 PMC2120709

[DMM048901C15] Filvaroff, E., Erlebacher, A., Ye, J., Gitelman, S. E., Lotz, J., Heillman, M. and Derynck, R. (1999). Inhibition of TGF-beta receptor signaling in osteoblasts leads to decreased bone remodeling and increased trabecular bone mass. *Development* 126, 4267-4279. 10.1242/dev.126.19.4267 10477295

[DMM048901C16] Fuchs, H., Sabrautzki, S., Przemeck, G. K., Leuchtenberger, S., Lorenz-Depiereux, B., Becker, L., Rathkolb, B., Horsch, M., Garrett, L., Ostereicher, M. A. et al. (2016). The First Scube3 Mutant Mouse Line with Pleiotropic Phenotypic Alterations. *G3 (Bethesda)* 6, 4035-4046. 27815347 10.1534/g3.116.033670 PMC5144972

[DMM048901C17] Gao, X., Gordon, D., Zhang, D., Browne, R., Helms, C., Gillum, J., Weber, S., Devroy, S., Swaney, S., Dobbs, M. et al. (2007). CHD7 gene polymorphisms are associated with susceptibility to idiopathic scoliosis. *Am. J. Hum. Genet.* 80, 957-965. 10.1086/513571 17436250 PMC1852746

[DMM048901C18] Grauers, A., Einarsdottir, E. and Gerdhem, P. (2016). Genetics and pathogenesis of idiopathic scoliosis. *Scoliosis Spinal Disord* 11, 45. 10.1186/s13013-016-0105-8 27933320 PMC5125035

[DMM048901C19] Gray, R. S., Gonzalez, R., Ackerman, S. D., Minowa, R., Griest, J. F., Bayrak, M. N., Troutwine, B., Canter, S., Monk, K. R., Sepich, D. S. et al. (2020). Postembryonic screen for mutations affecting spine development in zebrafish. *Dev. Biol.* 471, 18-33. 10.1016/j.ydbio.2020.11.009 33290818 PMC10785604

[DMM048901C20] Grewal, P. K., Holzfeind, P. J., Bittner, R. E. and Hewitt, J. E. (2001). Mutant glycosyltransferase and altered glycosylation of alpha-dystroglycan in the myodystrophy mouse. *Nat. Genet.* 28, 151-154. 10.1038/88865 11381262

[DMM048901C21] Henke, K., Daane, J. M., Hawkins, M. B., Dooley, C. M., Busch-Nentwich, E. M., Stemple, D. L. and Harris, M. P. (2017). Genetic Screen for Postembryonic Development in the Zebrafish (Danio rerio): Dominant Mutations Affecting Adult Form. *Genetics* 207, 609-623. 10.1534/genetics.117.300187 28835471 PMC5629327

[DMM048901C22] Herring, J. A. (2013). *Tachdjian's Pediatic Orthopaedics*. Philadelphia: WB Saunders.

[DMM048901C23] Hubaud, A. and Pourquie, O. (2014). Signalling dynamics in vertebrate segmentation. *Nat. Rev. Mol. Cell Biol.* 15, 709-721. 10.1038/nrm3891 25335437

[DMM048901C24] Inamori, K., Yoshida-Moriguchi, T., Hara, Y., Anderson, M. E., Yu, L. and Campbell, K. P. (2012). Dystroglycan function requires xylosyl- and glucuronyltransferase activities of LARGE. *Science* 335, 93-96. 10.1126/science.1214115 22223806 PMC3702376

[DMM048901C25] Karczewski, K. J., Francioli, L. C., Tiao, G., Cummings, B. B., Alfoldi, J., Wang, Q., Collins, R. L., Laricchia, K. M., Ganna, A., Birnbaum, D. P. et al. (2020). The mutational constraint spectrum quantified from variation in 141,456 humans. *Nature* 581, 434-443. 10.1038/s41586-020-2308-7 32461654 PMC7334197

[DMM048901C26] Karner, C. M., Long, F., Solnica-Krezel, L., Monk, K. R. and Gray, R. S. (2015). Gpr126/Adgrg6 deletion in cartilage models idiopathic scoliosis and pectus excavatum in mice. *Hum. Mol. Genet.* 24, 4365-4373. 10.1093/hmg/ddv170 25954032 PMC4492399

[DMM048901C27] Khanshour, A. M., Kou, I., Fan, Y., Einarsdottir, E., Makki, N., Kidane, Y. H., Kere, J., Grauers, A., Johnson, T. A., Paria, N. et al. (2018). Genome-wide meta-analysis and replication studies in multiple ethnicities identify novel adolescent idiopathic scoliosis susceptibility loci. *Hum. Mol. Genet.* 27, 3986-3998. 10.1093/hmg/ddy306 30395268 PMC6488972

[DMM048901C28] Kobayashi, T., Yamanaka, T., Jacobs, J. M., Teixeira, F. and Suzuki, K. (1980). The Twitcher mouse: an enzymatically authentic model of human globoid cell leukodystrophy (Krabbe disease). *Brain Res.* 202, 479-483. 10.1016/0006-8993(80)90159-6 7437911

[DMM048901C29] Korpal, M., Yan, J., Lu, X., Xu, S., Lerit, D. A. and Kang, Y. (2009). Imaging transforming growth factor-beta signaling dynamics and therapeutic response in breast cancer bone metastasis. *Nat. Med.* 15, 960-966. 10.1038/nm.1943 19597504

[DMM048901C30] Kou, I., Takahashi, Y., Johnson, T. A., Takahashi, A., Guo, L., Dai, J., Qiu, X., Sharma, S., Takimoto, A., Ogura, Y. et al. (2013). Genetic variants in GPR126 are associated with adolescent idiopathic scoliosis. *Nat. Genet.* 45, 676-679. 10.1038/ng.2639 23666238

[DMM048901C31] Lane, P. W., Beamer, T. C. and Myers, D. D. (1976). Myodystrophy, a new myopathy on chromosome 8 of the mouse. *J. Hered.* 67, 135-138. 10.1093/oxfordjournals.jhered.a108687 939913

[DMM048901C32] Lin, Y. C., Roffler, S. R., Yan, Y. T. and Yang, R. B. (2015). Disruption of Scube2 Impairs Endochondral Bone Formation. *J. Bone Miner. Res.* 30, 1255-1267. 10.1002/jbmr.2451 25639508

[DMM048901C33] Lin, Y. C., Niceta, M., Muto, V., Vona, B., Pagnamenta, A. T., Maroofian, R., Beetz, C., van Duyvenvoorde, H., Dentici, M. L., Lauffer, P. et al. (2021). SCUBE3 loss-of-function causes a recognizable recessive developmental disorder due to defective bone morphogenetic protein signaling. *Am. J. Hum. Genet.* 108, 115-133. 10.1016/j.ajhg.2020.11.015 33308444 PMC7820739

[DMM048901C34] Lorenz, C., Meyer-Kleine, C., Steinmeyer, K., Koch, M. C. and Jentsch, T. J. (1994). Genomic organization of the human muscle chloride channel CIC-1 and analysis of novel mutations leading to Becker-type myotonia. *Hum. Mol. Genet.* 3, 941-946. 10.1093/hmg/3.6.941 7951242

[DMM048901C35] Martin, P. T. (2003). Dystroglycan glycosylation and its role in matrix binding in skeletal muscle. *Glycobiology* 13, 55R-66R. 10.1093/glycob/cwg076 12736199

[DMM048901C36] Meyer-Kleine, C., Steinmeyer, K., Ricker, K., Jentsch, T. J. and Koch, M. C. (1995). Spectrum of mutations in the major human skeletal muscle chloride channel gene (CLCN1) leading to myotonia. *Am. J. Hum. Genet.* 57, 1325-1334. 8533761 PMC1801423

[DMM048901C37] Mizuguchi, T., Collod-Beroud, G., Akiyama, T., Abifadel, M., Harada, N., Morisaki, T., Allard, D., Varret, M., Claustres, M., Morisaki, H. et al. (2004). Heterozygous TGFBR2 mutations in Marfan syndrome. *Nat. Genet.* 36, 855-860. 10.1038/ng1392 15235604 PMC2230615

[DMM048901C38] Onyenwoke, R. U. and Brenman, J. E. (2015). Lysosomal storage diseases-regulating neurodegeneration. *J. Exp. Neurosci.* 9, 81-91. 10.4137/JEN.S25475 PMC4822725 27081317

[DMM048901C39] Ran, F. A., Hsu, P. D., Wright, J., Agarwala, V., Scott, D. A. and Zhang, F. (2013). Genome engineering using the CRISPR-Cas9 system. *Nat. Protoc.* 8, 2281-2308. 10.1038/nprot.2013.143 24157548 PMC3969860

[DMM048901C40] Rios, J. J., Denton, K., Russell, J., Kozlitina, J., Ferreira, C. R., Lewanda, A. F., Mayfield, J. E., Moresco, E., Ludwig, S., Tang, M. et al. (2021). Germline saturation mutagenesis induces skeletal phenotypes in mice. *J. Bone Miner. Res*. (in press). 10.1002/jbmr.4323 PMC8862308 33905568

[DMM048901C41] Sakai, N., Inui, K., Fujii, N., Fukushima, H., Nishimoto, J., Yanagihara, I., Isegawa, Y., Iwamatsu, A. and Okada, S. (1994). Krabbe disease: isolation and characterization of a full-length cDNA for human galactocerebrosidase. *Biochem. Biophys. Res. Commun.* 198, 485-491. 10.1006/bbrc.1994.1071 8297359

[DMM048901C42] Sharma, S., Gao, X., Londono, D., Devroy, S. E., Mauldin, K. N., Frankel, J. T., Brandon, J. M., Zhang, D., Li, Q. Z., Dobbs, M. B. et al. (2011). Genome-wide association studies of adolescent idiopathic scoliosis suggest candidate susceptibility genes. *Hum. Mol. Genet.* 20, 1456-1466. 10.1093/hmg/ddq571 21216876 PMC3049353

[DMM048901C43] Sharma, S., Londono, D., Eckalbar, W. L., Gao, X., Zhang, D., Mauldin, K., Kou, I., Takahashi, A., Matsumoto, M., Kamiya, N. et al. (2015). A PAX1 enhancer locus is associated with susceptibility to idiopathic scoliosis in females. *Nat. Commun.* 6, 6452. 10.1038/ncomms7452 25784220 PMC4365504

[DMM048901C44] Skalova, D., Zidkova, J., Vohanka, S., Mazanec, R., Musova, Z., Vondracek, P., Mrazova, L., Kraus, J., Reblova, K. and Fajkusova, L. (2013). CLCN1 mutations in Czech patients with myotonia congenita, in silico analysis of novel and known mutations in the human dimeric skeletal muscle chloride channel. *PLoS ONE* 8, e82549. 10.1371/journal.pone.0082549 24349310 PMC3859631

[DMM048901C45] Sparrow, D. B., Guillen-Navarro, E., Fatkin, D. and Dunwoodie, S. L. (2008). Mutation of Hairy-and-Enhancer-of-Split-7 in humans causes spondylocostal dysostosis. *Hum. Mol. Genet.* 17, 3761-3766. 10.1093/hmg/ddn272 18775957

[DMM048901C46] Sparrow, D. B., Sillence, D., Wouters, M. A., Turnpenny, P. D. and Dunwoodie, S. L. (2010). Two novel missense mutations in HAIRY-AND-ENHANCER-OF-SPLIT-7 in a family with spondylocostal dysostosis. *Eur. J. Hum. Genet.* 18, 674-679. 10.1038/ejhg.2009.241 20087400 PMC2987349

[DMM048901C47] Takahashi, Y., Kou, I., Takahashi, A., Johnson, T. A., Kono, K., Kawakami, N., Uno, K., Ito, M., Minami, S., Yanagida, H. et al. (2011). A genome-wide association study identifies common variants near LBX1 associated with adolescent idiopathic scoliosis. *Nat. Genet.* 43, 1237-1240. 10.1038/ng.974 22019779

[DMM048901C48] Tappino, B., Biancheri, R., Mort, M., Regis, S., Corsolini, F., Rossi, A., Stroppiano, M., Lualdi, S., Fiumara, A., Bembi, B. et al. (2010). Identification and characterization of 15 novel GALC gene mutations causing Krabbe disease. *Hum. Mutat.* 31, E1894-E1914. 10.1002/humu.21367 20886637 PMC3052420

[DMM048901C49] Tu, C. F., Yan, Y. T., Wu, S. Y., Djoko, B., Tsai, M. T., Cheng, C. J. and Yang, R. B. (2008). Domain and functional analysis of a novel platelet-endothelial cell surface protein, SCUBE1. *J. Biol. Chem.* 283, 12478-12488. 10.1074/jbc.M705872200 18303018

[DMM048901C50] van Reeuwijk, J., Grewal, P. K., Salih, M. A., Beltran-Valero de Bernabe, D., McLaughlan, J. M., Michielse, C. B., Herrmann, R., Hewitt, J. E., Steinbrecher, A., Seidahmed, M. Z. et al. (2007). Intragenic deletion in the LARGE gene causes Walker-Warburg syndrome. *Hum. Genet.* 121, 685-690. 10.1007/s00439-007-0362-y 17436019 PMC1914248

[DMM048901C51] Wang, T., Zhan, X., Bu, C. H., Lyon, S., Pratt, D., Hildebrand, S., Choi, J. H., Zhang, Z., Zeng, M., Wang, K. W. et al. (2015). Real-time resolution of point mutations that cause phenovariance in mice. *Proc. Natl. Acad. Sci. U.S.A.* 112, E440-E449. 10.1073/pnas.1423216112 25605905 PMC4321302

[DMM048901C52] Wang, T., Bu, C. H., Hildebrand, S., Jia, G., Siggs, O. M., Lyon, S., Pratt, D., Scott, L., Russell, J., Ludwig, S. et al. (2018). Probability of phenotypically detectable protein damage by ENU-induced mutations in the Mutagenetix database. *Nat. Commun.* 9, 441. 10.1038/s41467-017-02806-4 29382827 PMC5789985

[DMM048901C53] Watkins, W. J. and Watts, D. C. (1984). Biological features of the new A2G--adr mouse mutant with abnormal muscle function. *Lab. Anim.* 18, 1-6. 10.1258/002367784780865036 10628777

[DMM048901C54] Wenger, D. A., Rafi, M. A., Luzi, P., Datto, J. and Costantino-Ceccarini, E. (2000). Krabbe disease: genetic aspects and progress toward therapy. *Mol. Genet. Metab.* 70, 1-9. 10.1006/mgme.2000.2990 10833326

[DMM048901C55] Wise, C. A., Sepich, D., Ushiki, A., Khanshour, A. M., Kidane, Y. H., Makki, N., Gurnett, C. A., Gray, R. S., Rios, J. J., Ahituv, N. et al. (2020). The cartilage matrisome in adolescent idiopathic scoliosis. *Bone Res* 8, 13. 10.1038/s41413-020-0089-0 PMC7062733 32195011

[DMM048901C56] Wu, B. T., Su, Y. H., Tsai, M. T., Wasserman, S. M., Topper, J. N. and Yang, R. B. (2004). A novel secreted, cell-surface glycoprotein containing multiple epidermal growth factor-like repeats and one CUB domain is highly expressed in primary osteoblasts and bones. *J. Biol. Chem.* 279, 37485-37490. 10.1074/jbc.M405912200 15234972

[DMM048901C57] Wu, Y. Y., Peck, K., Chang, Y. L., Pan, S. H., Cheng, Y. F., Lin, J. C., Yang, R. B., Hong, T. M. and Yang, P. C. (2011). SCUBE3 is an endogenous TGF-beta receptor ligand and regulates the epithelial-mesenchymal transition in lung cancer. *Oncogene* 30, 3682-3693. 10.1038/onc.2011.85 21441952

[DMM048901C58] Wu, N., Ming, X., Xiao, J., Wu, Z., Chen, X., Shinawi, M., Shen, Y., Yu, G., Liu, J., Xie, H. et al. (2015). TBX6 null variants and a common hypomorphic allele in congenital scoliosis. *N. Engl. J. Med.* 372, 341-350. 10.1056/NEJMoa1406829 25564734 PMC4326244

[DMM048901C59] Wu, M., Chen, G. and Li, Y. P. (2016). TGF-beta and BMP signaling in osteoblast, skeletal development, and bone formation, homeostasis and disease. *Bone Res* 4, 16009. 10.1038/boneres.2016.9 27563484 PMC4985055

[DMM048901C60] Xavier, G. M., Panousopoulos, L. and Cobourne, M. T. (2013). Scube3 is expressed in multiple tissues during development but is dispensable for embryonic survival in the mouse. *PLoS ONE* 8, e55274. 10.1371/journal.pone.0055274 23383134 PMC3558425

[DMM048901C61] Yang, Y., Muzny, D. M., Xia, F., Niu, Z., Person, R., Ding, Y., Ward, P., Braxton, A., Wang, M., Buhay, C. et al. (2014). Molecular findings among patients referred for clinical whole-exome sequencing. *JAMA* 312, 1870-1879. 10.1001/jama.2014.14601 25326635 PMC4326249

[DMM048901C62] Yang, N., Wu, N., Zhang, L., Zhao, Y., Liu, J., Liang, X., Ren, X., Li, W., Chen, W., Dong, S. et al. (2019). TBX6 compound inheritance leads to congenital vertebral malformations in humans and mice. *Hum. Mol. Genet.* 28, 539-547. 10.1093/hmg/ddy358 30307510 PMC6489408

