# Supplementary material for: Saturation mutagenesis defines novel mouse models of severe spine deformity
Source: Dis Model Mech. 2021 Jun 18;14(6):dmm048901. doi: 10.1242/dmm.048901 (PMC8246263; doi:10.1242/dmm.048901)
Supplement: Supplementary information [file dmm-14-048901-s1.pdf]

Fig. S1

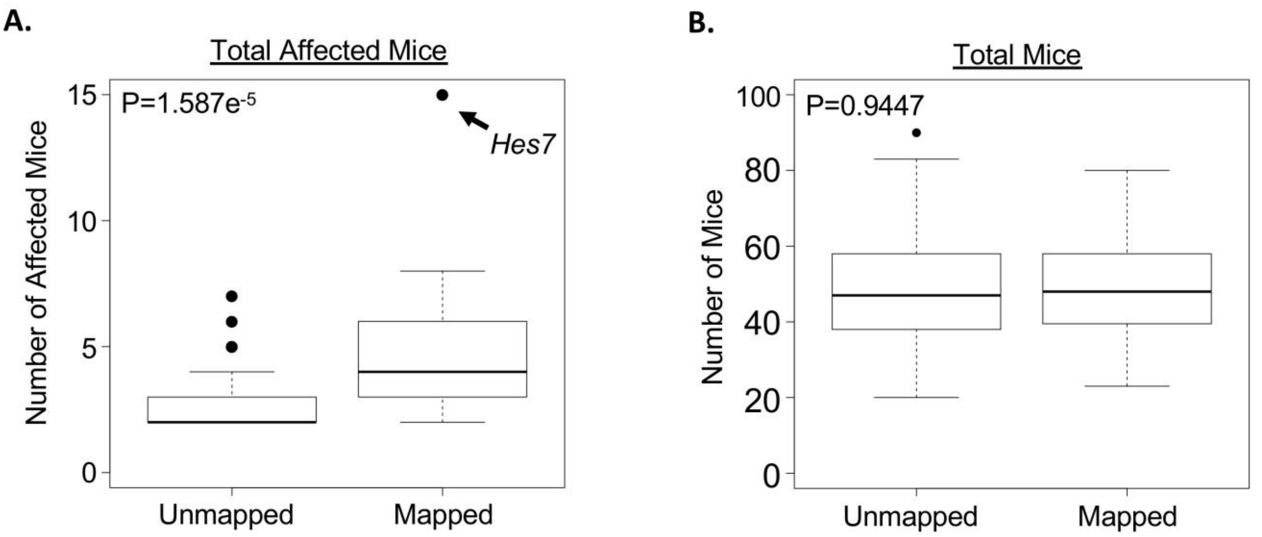

Fig. S1. Total number of affected mice is associated with successful meiotic mapping to ENU alleles. **(A,B)** The **(A)** total number of affected G3 mice in the pedigree and **(B)** total number of G3 mice in the pedigree were evaluated among 20 pedigrees with alleles mapped to a spine deformity phenotype compared to 74 pedigrees with at least two affected mice but for which no allele was mapped. The dominant mutations in *Hes7* is indicated. Statistically significant differences were determined using Wilcoxon test.

Disease Models & Mechanisms • Supplementary information

Fig. S2

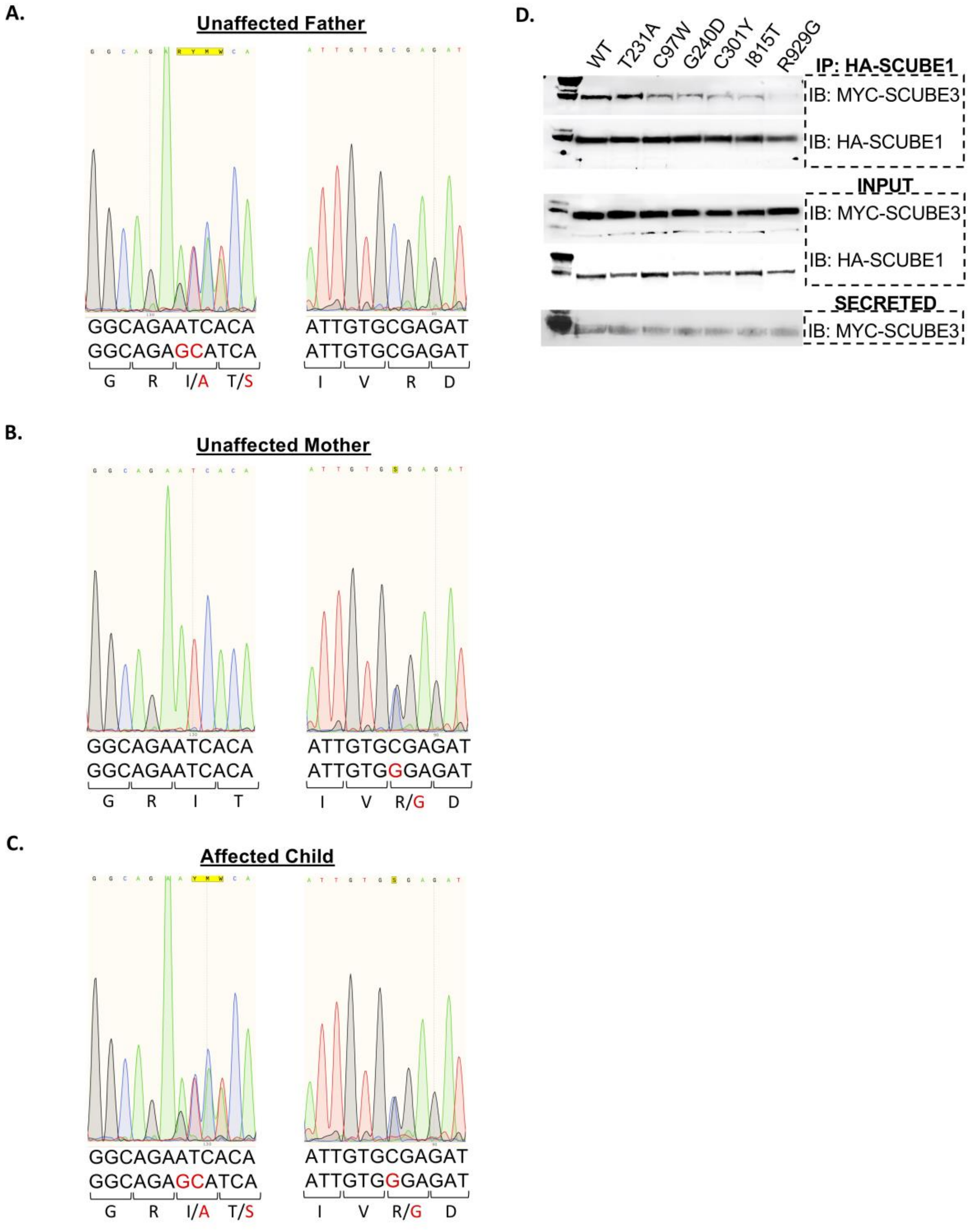

Disease Models & Mechanisms • Supplementary information

Fig. S2. Sanger sequence confirmation of *SCUBE3* mutations. **(A-C)** Sequence chromatograms testing presence of the *(left)* NM_152753.4: c.1521_1522insGC/p.(Ile508AlafsTer74) and (*right*) NM_152753.4: c.2785C>G/p.(Arg929Gly) mutations in the **(A)** unaffected father, **(B)** unaffected mother, and **(C)** affected patient. Resulting transcript and protein sequence is shown below, with mutant sequence shown in red. **(D)** Representative immunoblot (IB) of recombinant HA-tagged SCUBE1 and Myc-tagged SCUBE3 following immunoprecipitation (IP) of SCUBE1. Normal (WT) SCUBE3 and SCUBE3 harboring the common polymorphism (SCUBE3^{T231A}) are shown as positive controls. Input cell lysate is shown for both SCUBE1 and SCUBE3, and secreted SCUBE3 is also shown.

Fig. S3

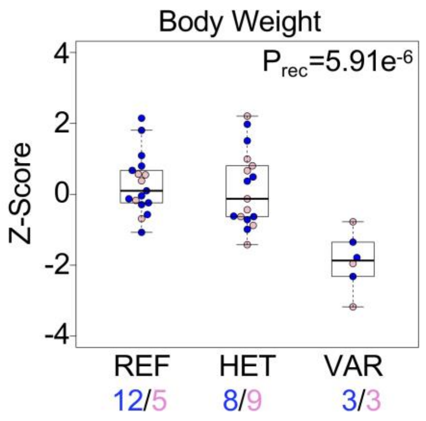

Fig. S3. The *Psma5*^{V146G} allele is associated with reduced body weight. Boxplot demonstrating the reduced body weight (adjusted Z-score) of mice homozygous for the *Psma5*^{V146G} allele (VAR) compared to mice heterozygous for the allele (HET) or homozygous for the reference allele (REF). Male mice are shown in blue and female mice shown in pink. Numbers of mice for each genotype are shown below. Statistical significance was determined by automated meiotic mapping for recessive alleles in the pedigree.

Fig. S4

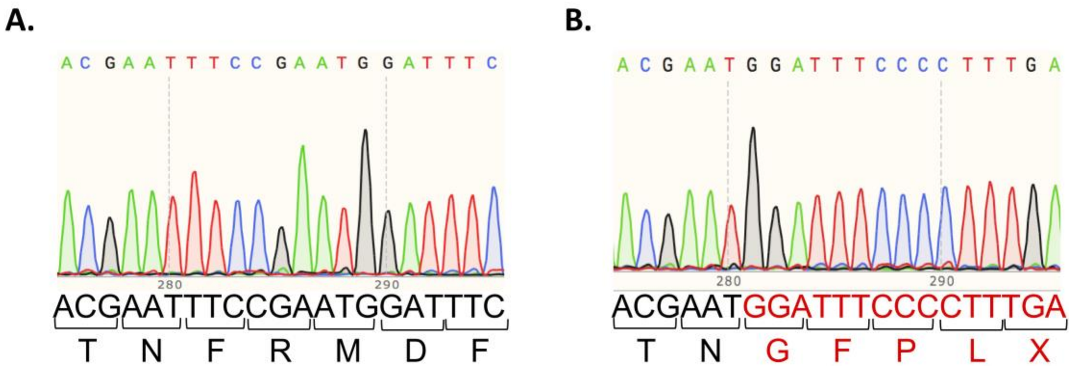

Fig. S4. Sequence confirmation of a loss-of-function allele in CRISPR-engineered *Clcn1* mice. **(A)** Representative chromatogram from a mouse homozygous for the wild-type allele. **(B)** Representative chromatogram from a mouse homozygous for the p.(Phe337GlyfsTer4) frameshift mutation. The deletion results in premature truncation, encoding a predicted protein of 336 amino acids. The wild-type protein has a length of 994 amino acids.

Disease Models & Mechanisms • Supplementary information

Tables S1 and S2.

Click here to download Tables S1 and S2

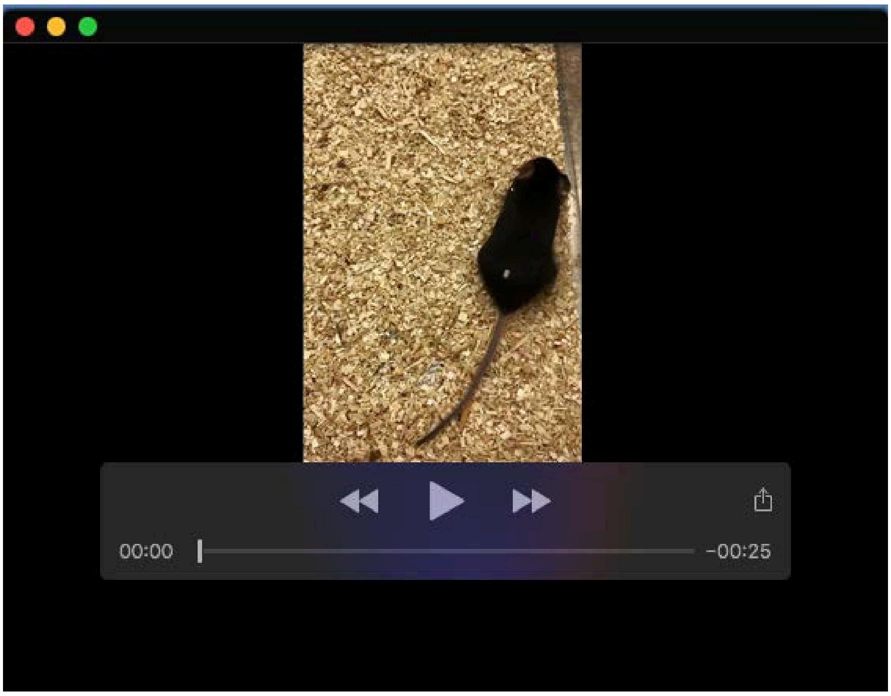

Movie 1. Altered gait in *Psma5*-mutant mice. Representative video of a 3-month old female mouse homozygous for the *Psma5*^{V146G} allele demonstrating the altered hindlimb gait and ataxia.

Disease Models & Mechanisms • Supplementary information
